# Nocturnal pain and fatigue in middle-aged persons with hip symptoms suspected to be osteoarthritis, is there a link in 10-year follow-up of the CHECK study?

**DOI:** 10.1016/j.ocarto.2023.100363

**Published:** 2023-04-14

**Authors:** Annemaria C. van Berkel, Dieuwke Schiphof, Jan H. Waarsing, Jos Runhaar, John M. van Ochten, Patrick J.E. Bindels, Sita M.A. Bierma-Zeinstra

**Affiliations:** a Department of General Practice, Erasmus MC University Medical Center Rotterdam, Rotterdam, the Netherlands; b Department of Orthopaedics, Erasmus MC University Medical Center Rotterdam, Rotterdam, the Netherlands

**Keywords:** Osteoarthritis, Hip, Cohort study, Fatigue, Nocturnal pain

## Abstract

**Objective:**

To explore the prevalence of nocturnal pain and fatigue in participants with hip symptoms suspected to be early osteoarthritis (OA) and to test the mediating effect of nocturnal pain on the association between hip OA pain and fatigue.

**Methods:**

We included participants with hip pain but no knee pain at baseline, from the Cohort Hip and Cohort Knee (CHECK)-study. Severity of hip OA pain was determined using the Numeric-Rating-Scale-pain-score last week. Fatigue was assessed using the SF-36 Fatigue subscale. Nocturnal pain was determined using the WOMAC-question: “How much pain have you experienced in the last 48 ​h at night while in bed?”. Hip OA pain, nocturnal pain and fatigue were measured repeatedly during 10-year follow-up. Path analysis were used per time point to determine the direct effect of OA pain on fatigue and the indirect effect through nocturnal pain.

**Results:**

In 170 participants (female: 76%; mean age: 55.7 years; mean BMI: 25.5 ​kg/m^2^) the prevalence of nocturnal pain varied between 22 and 35% and the prevalence of fatigue ranged between 14 and 18%. Hip OA pain was associated with nocturnal pain and fatigue. The direct effect of hip OA pain on fatigue was significant at all-time points. No significant mediating effect of nocturnal pain was found.

**Conclusion:**

In this cohort of participants suspected to have early hip OA, the prevalence of fatigue remained stable and the prevalence of nocturnal pain decreased slightly over 10-year follow-up. We did not find a mediating effect of nocturnal pain in the pathway between hip OA pain and fatigue.

## 1 Introduction

Osteoarthritis (OA) has a negative impact on general health, mental health, sleep, and overall energy [1]. Many patients with OA experience elevated levels of fatigue [2,3]. Most patients with hip OA experience chronic pain, disability, loss in function, and report nocturnal pain [4]. Intrusive nocturnal pain is used by surgeons (and other physicians) as a criterion for recommending total joint replacement [[5], [6], [7]]. While nocturnal pain is used as a reason to refer patients and opt for surgery, nocturnal pain is also reported as present regardless of the stage of OA [8]. Reports have shown a wide range in the prevalence of nocturnal hip pain in OA patients, ranging from 17% to 85% [8,9], Both nocturnal pain [8] and fatigue [3] were highlighted as a key concern by patients with hip OA.

Fatigue in OA is not routinely evaluated, but it is known that fatigue has an essential impact on patients’ lives [3]. Participants generally perceived fatigue as different from sleepiness and distinguished physical from mental fatigue [3]. Hawker et al., longitudinally demonstrated links between disability and depressed mood and fatigue, indicating that (OA) pain leads to depressed mood through its effects on fatigue and disability [10]. Nocturnal pain could also be a cause of fatigue, independent of average hip pain; for instance nocturnal pain could play a role in the pathway between severity in hip OA pain and fatigue. We hypothesize that nocturnal pain is a cause of fatigue based on observations that 1) nocturnal knee pain was shown to be related to sleep problems [11], and 2) poor sleep was significantly linked to fatigue among people with hip and knee OA [12].

**Fig. 1:**
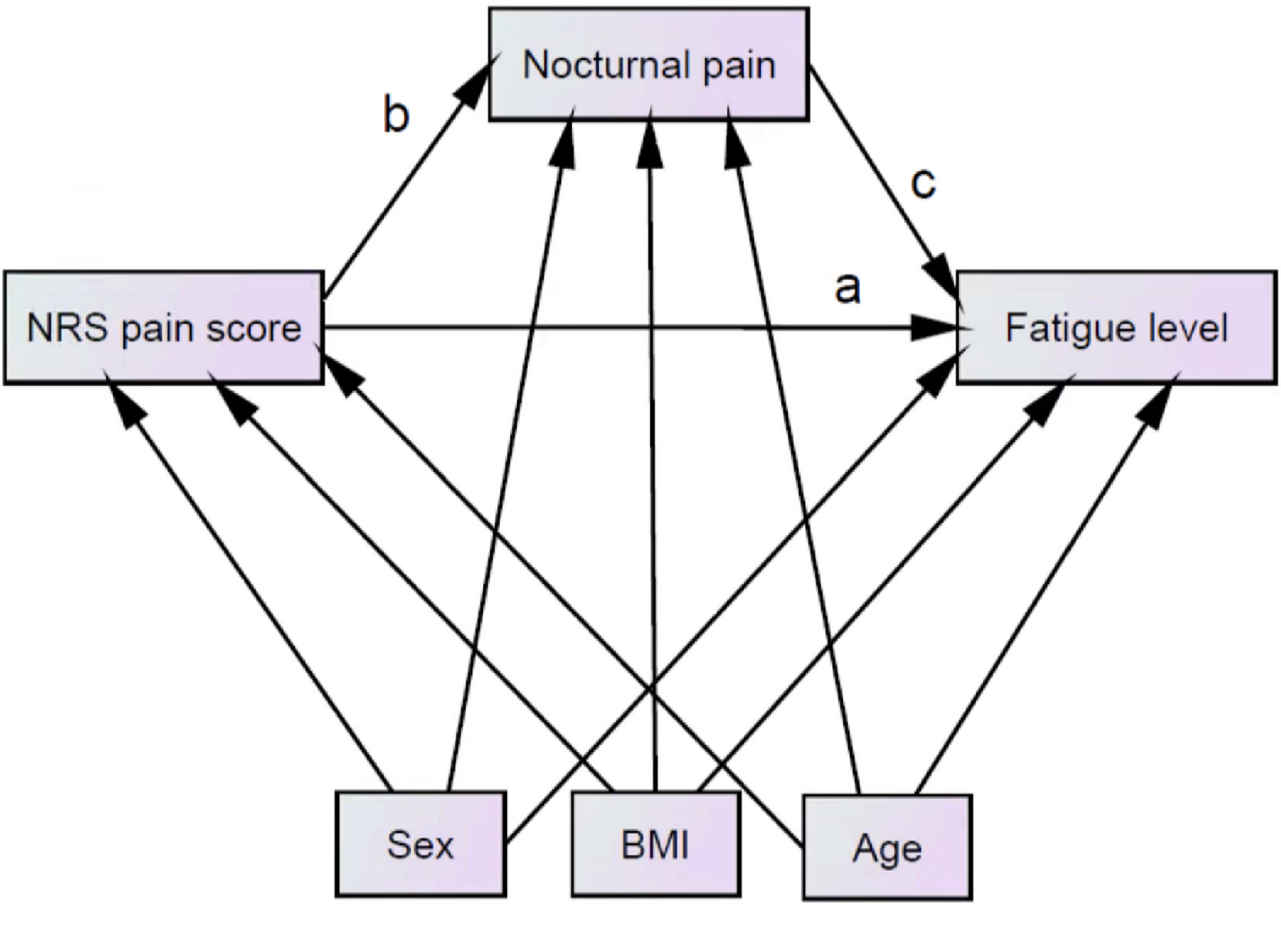
**Testing for the mediation effect of nocturnal pain on the cross-sectional association between hip OA pain (NRS pain score) and fatigue level, adjusted for confounders (sex, BMI, and age).** The total effect severity of hip OA pain includes the product of the direct effect (a) an the indirect effects (b, c). This model is applicable for baseline. The model can be applied in the same way for T2, T5, T8 and T10. The estimates are presented in Table 3 for the outcomes a, b and c. a ​= ​the association between NRS pain score and fatigue, with a regression weight for “a”, which means: when the NRS pain score goes up by 1, the fatigue score goes down by the value of the estimation “a” (when a negative estimation “a”) or the fatigue score goes up by the estimation “a” (when the value of the estimation “a” is a positive value). b ​= ​the association between NRS pain score and nocturnal pain, with a regression weight for “b”, which means: when the NRS pain score goes up by 1, the nocturnal pain goes up by the value of the estimation “b”. c ​= ​the association between nocturnal pain and fatigue, with a regression weight for “c”, which means: when the nocturnal pain score goes up by 1, the fatigue score goes down by the value of the estimation “c” (when a negative estimation “c”) or the fatigue score goes up by the value of the estimation “c” (when the estimation “c” is a positive value). A hypothetical model in which nocturnal pain acts as mediator in the pathway between hip OA pain severity and fatigue in individuals with hip OA. If the regression coefficients ‘b’ and/or ‘c’ are non-significant, there is no mediation. If the regression coefficients ‘a’ remains significant after adding the mediator ‘nocturnal pain’ to the model, then there is partial mediation. If regression coefficient ‘a’ is non-significant, but ‘b’ and ‘c’ are significant, then there is complete mediation. If regression coefficients ‘a’ and ‘b’ are significant, but ‘c’ is not, there is no mediation.

Fatigue in individuals with hip symptoms suspected to be early stage hip OA is essential to better understand the possible pathways leading to fatigue and to identify strategies to reduce the impact of the symptoms. Furthermore, more knowledge on nocturnal pain could be beneficial to enable healthcare professionals, especially general practitioners (GP) to educate patients in their early stage. The first aim of this study was to test the prevalence of nocturnal pain and fatigue in individuals with hip symptoms suspected to be early OA. The second aim was to test the hypothesis that nocturnal pain plays a role in the pathway between severity in hip OA pain and fatigue in individuals with hip symptoms suspected to be early OA (Fig. 1).

## 2 Methods

### 2.1 General design

The data for the present study were acquired from the Cohort Hip and Cohort Knee (CHECK) study; details on this cohort are published elsewhere [13], likewise the course of this study population [14]. To summarize, the CHECK study is a prospective, 10-year follow-up cohort in the Netherlands of 1002 individuals with hip and/or knee symptoms suspected to be early stage hip or knee OA [15]. The CHECK study is representative for the primary care and compared with another OA cohort [16]. Individuals entered the cohort in the period October 2002 to September 2005. The inclusion criteria were: having stiffness and/or pain of the hip and/or knee, aged between 45 and 65 years (relatively young age to increase the possibility of 10 years follow-up and to include the participants in their early stage of their disease), and not having yet consulted their GP for these symptoms, or having their first consultation within 6 months before the study entry. Exclusion criteria were having any other pathologic condition that could explain the symptoms (e.g., other rheumatic disease, previous hip/knee joint replacement, congenital dysplasia, osteochondritis dissecans, intra-articular fractures or injury, septic arthritis, (pseudo) radical syndrome etc.), having comorbidity that would not allow physical evaluation and/or follow-up over a period of at least 10 years, malignancy in the past five years, and inability to understand the Dutch language. For the present study we selected all participants who reported hip pain only (yes/no) (and no knee pain) at baseline. We define those participants as early hip OA, we need to note that we cannot be sure that hip OA is the source of ‘hip pain’ in all these cases. However, it is less likely that the pain is due to other diseases than hip OA. Medical ethics committees in all the participating centers [13] approved the study, and all participants gave written informed consent. Details for follow-up rate for each follow-up visit are published elsewhere [17].

### 2.2 Severity of hip OA pain (independent variable)

The severity of the symptoms suspected to be hip OA pain was determined using the Numeric Rating Scale (NRS) for pain during the past week (0-10), with a higher score indicating worse pain. The hip OA pain was assessed at all follow-up moments (at baseline, and after two years (T2), five years (T5), eight years (T8), and 10 years (T10)). At baseline and T2, participants were asked to score the pain they experienced in their most painful joint over the past week. From T5 onwards, participants were asked to score their pain related to the left and right hip separately. Of these measurements, we used the highest pain scores for the analyses.

### 2.3 Nocturnal pain (potential mediator)

The presence of nocturnal pain is measured in the Western Ontario and McMaster Universities Osteoarthritis Index (WOMAC) subscale pain score by the question “How much pain have you experienced in the last 48 ​h at night while in bed?” (possible responses: “none”, “mild”, “moderate”, “severe”, and “extreme”). Participants were classified as having nocturnal pain if they answered that they had “moderate”, “severe”, or “extreme” pain and were classified as not having nocturnal pain if they answered “none” or “mild”.

### 2.4 Fatigue (outcome variable/dependent variable)

Fatigue was determined using the SF-36 Fatigue subscale (also called Vitality subscale or Energy subscale) [18]. The SF-36 Fatigue subscale consists of four questions (“Did you feel full of pep?”; “Did you have a lot of energy?”; “Did you feel worn out?”; “Did you feel tired?”) evaluating the past four weeks. Each item has six response options ranging from “all of the time” to “none of the time”. The items are summed and linearly transformed to give a range from 0 to 100, with a higher score indicating better health, i.e., less fatigue. The SF-36 questionnaire was completed by participants at all follow-up moments.

Participants were classified as fatigued if their Fatigue subscale score was less than or equal to 45. This score corresponds to the 25th percentile in the U.S. general female population and was advised to use by Rose et al. [19,20].

### 2.5 Other baseline population characteristics

Further information was collected on pain and hip symptoms, physical functioning of the lower limb, medication use, alcohol use, comorbidities, Physically activity, quality of life, and psychosocial factors using (self-reported) questionnaires and physical examination at all five time points, details are published elsewhere [13]. The demographic variables used were age, sex, ethnicity, height and weight to calculate body mass index (BMI), and education level. The WOMAC questionnaire was also used to measure stiffness (0–8), physical functioning (0-68), and pain (0-20) and to give a total summed score (0–100), with a higher score indicating worse health.

Baseline standardized radiographs of the anteroposterior view (AP), pelvis view or unilateral faux profile view (FP) (both hips) of the hips were used to determine hip OA. Radiographs were centrally scored [21] for OA features according to the Kellgren and Lawrence (K&L) criteria [22]. In the hip, all radiograph features showed good inter-observer reliability (0.71–0.91) [23]. Radiographic OA (ROA) was defined as K&L grade ≥2.

### 2.6 Statistical analyses

Descriptive statistics and correlation analyses were performed with SPSS V24.0 (IBM). Prevalence was measured by the percentage and the number of participants with the outcome (e.g., nocturnal pain and fatigue; namely fatigue score of 45 or less) out of the total number of available participants; the total numbers varied during follow-up due to temporary or permanent loss to follow-up and missing data (Supplemental Fig. S1). Path analysis was used to estimate the association between the variables examined in our theoretical model. Path analysis was performed with AMOS V23.0 (IBM, SPSS), using maximum-likelihood estimation. In the model, hip OA pain was entered as a continuous independent variable, nocturnal pain was used as the hypothesized mediator and fatigue as the continuous outcome variable. Confounders included in the model were age, sex, and BMI. The BMI used in the model was that measured at the same time point as the hip OA pain. The model was tested cross-sectionally at baseline, T2, T5, T8, and T10. Causality models were built using path analysis to test the mediating effect of nocturnal pain independently over the 10-year follow-up. This analysis will be explorative because the measurement points are two or three years apart. This means that the independent variable (hip OA pain) in the model is the measured value at least one measurement moment before (time-1) the potential mediator (nocturnal pain) (time) and the outcome variable (fatigue) is the measured value at least one measurement moment after (time+1) the potential mediator in the model. Lastly, to test the hypothesis in a larger group and to test if in participants with hip pain combined with knee pain the results differed, we conducted the same analysis in all participants with hip pain (n ​= ​588). Prior to the analysis, the assumptions regarding multivariate normality and multicollinearity were tested. As recommended, different goodness-of-fit indices were used to estimate the model fit, namely the χ2, the Comparative Fit Index (CFI), and the root mean square error of approximation (RMSEA). A model was assumed to have a good fit if the ratio of χ2 to the degrees of freedom (χ2/df) was less than 3.0 and the CFI was larger than 0.90 [24]. RMSEA values <0.06 were considered ideal and values between 0.08 and 0.10 were considered acceptable [25]. Hoelter's critical N is the largest sample size for which one would accept the hypothesis that a model is correct. Effects were deemed to be statistically significant when P ​< ​0.05.

## 3 Results

### 3.1 Baseline measurements

Of the 1002 participants of the CHECK study, 170 participants reported only hip pain and were therefore included in our analyses. An overview of their baseline characteristics is shown in Table 1. Of the 170 participants, the majority were female (76%). The 170 participants had a mean age of 55.7 (SD 5.6) years and a mean BMI of 25.5 (SD 3.5) kg/m^2^.

**Table 1:** Baseline characteristics of the study population.

Baseline characteristics	
Number of participants	170
Age in years, mean (SD)	55.7 (5.6)
Female, n (%)	129 (76)
Body Mass Index (kg/m^2^), mean (SD)^a^	25.5 (3.5)
Highest education level (higher education), n (%)^a^	65 (39)
Use of any pain medication, n (%)^a^	63 (38)
No use of alcohol, n (%)^a^	30 (18)
Three or more comorbidities, n (%)^a^	33 (20)
Physically active (>30 ​min) for three or more times a week, n (%)^a^	103 (62)
Nocturnal pain, n (%)^a^	57 (35)
SF-36 fatigue subscale (0–100), mean (SD)^a^	65.3 (17.0)
Fatigued^±,^^a^	30 (18)
Baseline NRS (0-10) in past week, mean (SD)^a^	3.4 (2.2)
**WOMAC, mean (SD)**^a^	
• Pain (0-20)	4.8 (3.2)
• Stiffness (0–8)	2.5 (1.7)
• Physical function (0-68)	14.7 (11.1)
• Total sum score (0–100)	22.6 (15.4)
Radiographic severity K/L grade ≥2 either hip, n (%)^a^	38 (23)

Mean values (standard deviation), or numbers (percentages %). NRS= Numeric Rating Scale (0-10).

WOMAC = Western Ontario and McMaster osteoarthritis index. K/L ​= ​Kellgren and Lawrence score.

± ​= ​SF-36 subscale scored ≤45.

a Missing values ​< ​4%.

### 3.2 Course of hip OA pain, nocturnal pain, and fatigue over 10 years

At baseline, the prevalence of nocturnal pain was 35% (57 out 165 participants), the prevalence of fatigue was 18% (30 out 167 participants), and the mean Fatigue score was 65.3 (SD 17.0) (Table 2).

**Table 2:** Course during follow-up of hip OA pain, nocturnal pain, Fatigue score, and fatigued participants.

	Baseline	T2	T5	T8	T10
Hip OA pain (0-10), mean (SD)	3.4 (2.2)	3.0 (2.2)	3.0 (2.6)	2.3 (2.3)	2.5 (2.5)
Nocturnal pain, n yes/total participants (%)	57/165 (35)	42/154 (27)	38/147 (26)	31/143 (22)	33/139 (24)
SF-36 fatigue subscale (0–100), mean (SD)	65.3 (17.0)	64.8 (17.2)	66.1 (17.6)	66.4 (17.2)	66.4 (18.5)
Fatigued, n yes/total participants (%)	30/167 (18)	22/156 (14)	22/150 (15)	24/144 (17)	24/142 (17)

Mean values ​± ​the standard deviation, or number of participants with outcome/total (available) participants and the percentages %.

The mean NRS pain score indicating hip OA pain during follow-up ranged from 3.4 (SD 2.2) to 2.3 (SD 2.3) (Table 2); the score during follow-up decreased slightly. During follow-up, the mean score for the Fatigue scale ranged from 64.8 (SD 17.2) to 66.4 (SD 18.5) (Table 2). The prevalence of fatigued participants during follow-up varied between 14% and 18%. The percentage of participants who were fatigued at all time points was 4% (5 out of 142 participants). Table 2 shows that the prevalence of nocturnal pain decreased slightly, the prevalence ranged during follow-up from 35% to 22%; 5% (7 out of 139 participants) reported nocturnal pain at all time points during the follow-up period.

### 3.3 Path analysis

The direct effect of hip OA pain on fatigue (continuous variable) was statistically significant at all time points. For example, the baseline association between hip OA pain and fatigue was statistically significant (β ​= ​−1.44, P ​= ​0.01), indicating that a higher OA hip pain score is associated with more fatigue: when the NRS pain OA-score goes up by 1, the fatigue score goes down by 1,44. Similar direct associations were found at T2, T5, T8, and T10 (Supplemental Table S1). The OA hip pain showed significant positive associations with nocturnal pain (cross-sectional analysis for all time points), indicating that higher levels of hip OA pain were associated with the presence of nocturnal pain. The model used in path analysis for testing the mediating effect of hip OA pain on fatigue through nocturnal pain is depicted in Fig. 1. There was no statistically significant indirect effect of hip OA pain on fatigue (as a continuous variable) via nocturnal pain. The direct effect of hip OA pain on nocturnal pain was statistically significant (for baseline: β ​= ​0.08, P=<0.01); however, the effect of nocturnal pain on fatigue was not statistically significant (β ​= ​−1.46, P ​= ​0.60) (Table 3). Similar associations were found at T2, T5, T8, and T10 (Table 3). No significant mediating effect of nocturnal pain between hip OA pain and fatigue was found. Similarly, no mediating effect was found in the model used for longitudinal analyses (Supplementary Table S2 and Supplementary Fig. S1). To address the potential bias due to hip replacements, we tested a model-based without participants who received a hip replacement, which did not show significant differences (Supplementary Table S3). Supplementary Tables S4–5 showed the results if we included all participants with hip pain. Increasing the study-population, we found mediating effects, cross-sectional from 5 years follow-up forwards and for longitudinal analysis for most of the models.

**Table 3:** Results from testing the mediating effect of nocturnal pain on the association between hip OA pain and fatigue (continuous variable) adjusted for age, sex, and BMI. The estimations are presented with p-value and the 95% confidence intervals.

Model (n ​= ​170)	Regression weight for a (*p*-value) (95% CI)	Regression weight for b (*p*-value) (95% CI)	Regression weight for c (*p*-value) (95% CI)	χ^2^/df	RMSEA	CFI	Hoelter 0.05 index
Baseline	−1.33 (p ​= ​0.03) (−2.53, −0.13)	0.08 (p ​< ​0.01) (0.04, 0.12)	−1.46 (p ​= ​0.60) (−6.97, 4.05)	1.99/3	<0.01	1.00	665
T2	−1.87 (p ​< ​0.01) (−3.16, −0.58)	0.08 (p ​< ​0.01) (0.04, 0.12)	1.01 (p ​= ​0.76) (−5.34, 7.42)	3.21/3	0.02	1.00	411
T5	−1.45 (p ​= ​0.02) (−2.65, −0.25)	0.08 (p ​< ​0.01) (0.06, 0.10)	−6.34 (p ​= ​0.08) (−13.49, 0.81)	3.28/3	0.02	1.00	403
T8	−1.75 (p ​= ​0.01) (−3.04, −0.46)	0.06 (p ​< ​0.01) (0.04, 0.08)	−2.92 (p ​= ​0.42) (−10.02, 4.18)	2.93/3	<0.01	1.00	452
T10	−1.52 (p ​= ​0.02) (−2.79, −0.25)	0.07 (p ​< ​0.01) (0.05, 0.09)	−4.47 (p ​= ​0.25) (−12.07, 3.13)	4.53/3	0.05	0.97	292

## 4 Discussion

In this study focusing on participants with hip pain suspected to be early-stage hip OA, we showed that the prevalence of nocturnal pain over the 10-year follow-up ranged between 22% and 35%. The prevalence of fatigued participants ranged between 14% and 18% during the 10-year follow-up. Overall, the results showed that nocturnal pain was not associated with fatigue, but hip OA pain was associated with fatigue during the entire 10-year period. Hip OA pain severity was also positively associated with nocturnal pain. Our findings did not show that nocturnal pain mediated the significant association between hip OA pain and fatigue.

To our knowledge, fatigue combined with nocturnal pain in participants with hip OA have rarely been described in the literature. In our group of participants with hip symptoms suspected to be early hip OA, we found that nocturnal pain was present in a substantial proportion of them (35% at baseline). Patients often say that nocturnal pain is a key concern and it is also used as an indicator by physicians for hip replacement [[5], [6], [7]]. Studies have shown better quality of life and less fatigue (SF-36) as well as reduced pain after total joint replacement [[26], [27], [28]]. However, as far as we know, there is little known about the improvement of nocturnal pain after hip replacement. Another study showed that the satisfaction in patients who underwent joint replacement was not influenced by the presence of pre-operative nocturnal pain [29]. This, combined with our result of no association between nocturnal pain and fatigue, raises the question of how well nocturnal pain has been studied as an indicator for hip replacement.

Our study obtained a prevalence for nocturnal pain varying between 22% and 35%, during the 10 years of follow-up. This is in line with Hawker et al. who reported that 17% of hip OA patients reported nocturnal pain using the Patient Generated Index (PGI) [9]. Woolhead et al. described the same population, but found that 85% of the participants with hip OA in the focus groups reported nocturnal pain [8]. These participants were selected from the community as well from existing OA cohorts and were included if they had hip or knee OA. Compared to our study group, the study population in the focus groups was smaller, reported overall higher WOMAC scores and higher pain levels, and was on average older (by 13 years) [8,9]. In a previous paper we showed that the WOMAC score overall in our population was stable during the follow-up [14]. Our study is focused more on participants in primary care in an early stage of their OA, which might also explain the difference in nocturnal pain prevalence. Furthermore, the way nocturnal pain is defined is of huge influence on the prevalence. With the PGI, the patient identifies the most important symptoms or pain characteristics that they consider most distressing [9], which is a different strategy to discussing nocturnal pain in a focus group or answering a question about nocturnal pain in a questionnaire. The focus of this paper was predominantly on hip OA. In another paper we showed the contrast between people with hip and knee OA and nocturnal pain [30]. In this paper we found that nocturnal pain was reported more often by participants with hip OA compared to participants with knee OA. However, this difference was not significant. In a paper in people with radiographic knee OA, they showed that the prevalence of nocturnal knee symptoms was correlated the presence of central sensitization [31]. This might also be a possibility for people with hip OA, but more research is needed to compare knee and hip pain and nocturnal pain.

Fatigue in patients with OA is not often evaluated and has only been considered in a limited number of studies [3,[32], [33], [34], [35], [36]]. In addition, previous studies have focused primarily on patients with OA in secondary care, who form a minority of people with OA [3], or the studies are based on results obtained from focus groups [32] or also include participants with other diseases like rheumatoid arthritis or fibromyalgia [[34], [35], [36]]. Wolfe et al. reported that 41% of patients with hip/knee/hand OA have substantial fatigue [32]. Compared to our study, the participants in this study had on average a higher BMI, were older, and more importantly, included all the participants from a rheumatologic clinic, whereas our study focused on participants in primary care in an early stage of their OA [32]. The prevalence of fatigue in our study was lower (ranged between 14% and 18% during follow-up) compared to Wolfe et al. [32], Fatigue trajectories in another study using CHECK data concluded that a considerable number of participants, with both hip and knee symptoms, already experience elevated levels of fatigue at an early stage of OA [2]. The majority of the studies mentioned above that examined fatigue did not provide an estimate of the prevalence of fatigue. Again, the way fatigue is defined is of huge influence. There are studies that measured fatigue as a scale with one single question, while other studies used a questionnaire with multiple questions (like our present study). Moreover, various questionnaires are used to evaluate fatigue, whereby some provide a continuous scale only while others also provide classes (e.g., normal fatigue, moderate fatigue, and severe fatigue).

In this article we hypothesized that nocturnal pain plays a role in the pathway, i.e., acts as a mediating factor between hip OA pain and fatigue in participants with hip pain suspected to be hip OA. This hypothesis was based on the following evidence: 1) nocturnal knee pain was shown to be related to sleep problems [11]; 2) poor sleep is significantly linked with fatigue among people with OA [12]. We did not find a significant mediation effect. This might be due to the way we defined nocturnal pain: we used the WOMAC question, which could be too general. A more direct question could be preferable, for example: “At night, do you experience pain in your hip? If yes, do you think it affects your sleep?” Or: “Does the pain in your hip awaken you from your sleep (during the night)?” Also making a binary variable of the WOMAC nocturnal pain question could have played a role. Another reason could be that we lacked information on the quality of sleep. We know sleeping disorders affect the level of fatigue. Those sleep disorders are common in older people, in women, and in people with higher BMI [37], the same groups in which OA is prevalent.

In principle longitudinal data is required to test for causality (and mediation). Our CHECK cohort with its longitudinal design allowed for a mediation analysis whereby exposure was measured two (or three) years before the mediator, and the mediator was assessed two (or three) years before the outcome of interest. These analyses (Supplementary Table S1) did not show any consistent significant mediation effects. Data that are closer together in time (e.g., a few weeks or months apart) would be more informative and might demonstrate mediation. Our results suggest that the effect of the severity of hip OA pain on fatigue was not mediated through nocturnal pain.

A limitation of this study design is that despite correcting for known confounders, we still could have had unmeasured confounding that influenced the estimates of direct and indirect effects, e.g., mental health, depression, lack of sleep/quality of sleep. There were also a few possible confounders available in our data, we did not included. For example radiographic severity (radiographic hip OA); based on research about the discordance between radiographic OA and clinical symptoms [38], in addition to the early stage of hip OA in the present population we do not believe the radiographic severity will change the results. Additional analysis for the hip group adding radiographic hip OA in any hip as an confounder did not change the results (see Supplementary Table S6). Menopausal status could also possibly influenced the estimates of direct and indirect effect, however, our model did not allow more confounders. We believe that adjusting with the two confounder sex and age, it will be sufficient for the menopausal status as well. Another limitation is the relatively small group of participants. We included only 170 of the 1002 participants of the CHECK study with solely hip pain, to be confident that the nocturnal pain was related to the hip joint. The size of our study population is not an issue for the estimates of prevalence, but the sample size might be a limitation for path analysis. A rule of thumb says that the minimum sample size should be equal to the number of parameters being estimating in the model multiplied by between 5 and 20. Several factors affect sample size requirement, namely the complexity of the model, the variables used, and the score reliability [24]. Our model is relatively simple with only six variables, which are mainly continuous and normally distributed; therefore our sample size should be sufficient. However, our study might be underpowered for longitudinal modelling. If we add the participants with hip and knee pain (n ​= ​588) into the analyses we found nocturnal pain as mediator in the cross-sectional analysis (>5 years of follow-up) and also longitudinally. Therefore, their might be a mediating effect of nocturnal pain, but more research is needed to disclose how big this mediating effect is. This study also has its strengths; it is one of the first studies providing long-term information about fatigue and nocturnal pain in participants early in their OA process/with hip pain suspected to be hip OA. As far as we know, this study is also the first study analyzing nocturnal pain as a mediator. The use of the CHECK study, a prospective, observational cohort study with a 10-year follow-up, is a strength because the study is centrally coordinated to guarantee high-quality data.

In conclusion, based on the results of this study, 22–35% of our study population reported nocturnal pain and 14–18% were classified as fatigued during the 10-year follow-up. Severity of hip OA pain was associated with both nocturnal pain and fatigue, but no evidence was found that nocturnal pain acted as a mediator. This means that in patients with hip pain suspected to be hip OA, treatment targeting nocturnal pain will probably not reduce fatigue, or only do so to a limited degree. However, both nocturnal pain and fatigue are concerns of patients, therefore more research is needed to provide more background on these features and to improve treatments. Nocturnal pain (especially in those who will receive a hip replacement) deserve further scrutiny, for example: could it be possible that chronic pain and fatigue share neurological mechanisms within the brain? Future research is also needed to examine if these findings are similar in bilateral hip OA.

## Authors' contributions

ACB, DS, SBZ contributed to the conception and design of this study. ACB, DS, JW and SBZ contributed to the analysis of data. All authors contributed to the interpretation of data. Article drafts were written by ACB and critically revised by all authors. The final version of the article was approved by all authors.

## Data availability statement

Datasets analyzed during the current study are available from the corresponding author on reasonable request.

## Role of funding

CHECK is funded by the Dutch 10.13039/501100000142Arthritis Society.

## Declaration of competing interest

Professor Sita receives consulting fees from Pfizer Infirst Healthcare.
