# Supplementary material for: Nocturnal pain and fatigue in middle-aged persons with hip symptoms suspected to be osteoarthritis, is there a link in 10-year follow-up of the CHECK study?
Source: Osteoarthr Cartil Open. 2023 Apr 14;5(3):100363. doi: 10.1016/j.ocarto.2023.100363 (PMC10192639; doi:10.1016/j.ocarto.2023.100363)
Supplement: Multimedia component 1 [file mmc1.docx]

**Nocturnal pain and fatigue in middle-aged persons with hip symptoms suspected to be osteoarthritis, is there a link in 10-year follow-up of the CHECK study?**

Supplementary material

A.C. van Berkel, MD^1^, D. Schiphof, PhD^1^, J.H. Waarsing, PhD^2^, J. Runhaar, PhD^1^, J.M. van Ochten, PhD^1^, P.J.E. Bindels PhD, professor^1^, S.M.A. Bierma-Zeinstra, PhD, professor^1,2^

1 Department of General Practice, Erasmus MC University Medical Center Rotterdam, Rotterdam, The Netherlands, 2 Department of Orthopaedics, Erasmus MC University Medical Center Rotterdam, Rotterdam, The Netherlands

**Supplementary Table S1** Results from the simple model for direct association between hip OA pain and fatigue (continuous variable) adjusted for age, sex, and BMI.

**Supplementary Table S2** Results from testing the longitudinal mediating effect of nocturnal pain on the association between hip OA pain and fatigue level.

**Supplementary Table S3** Results from testing the mediating effect of nocturnal pain on the association between hip OA pain and fatigue (continuous variable) adjusted for age, sex, and BMI for only those who did not received a hip replacement (n=132).

**Supplementary Table S4** Results from testing the mediating effect of nocturnal pain on the association between hip OA pain and fatigue (continuous variable) adjusted for age, sex, and BMI for those with hip pain (and possible knee pain n=588).

**Supplementary Table S5** Results from testing the longitudinal mediating effect of nocturnal pain on the association between hip OA pain and fatigue level for those with hip pain (and possible knee pain n=588).

**Supplementary Table S6** Results from testing the mediating effect of nocturnal pain on the association between hip OA pain and fatigue (continuous variable) adjusted for age, sex, Kellgren and Laurence score ≥2 any hip and BMI. The estimations are presented with p-value.

**Supplementary Figure S1** Flow-chart indicating the available data per time point.

**Supplementary Figure S2** Testing for the longitudinal mediating effect of nocturnal pain on the association between hip OA pain (NRS pain score) and fatigue level, adjusted for confounders (sex, BMI, and age).

**Supplementary Figure S3** Scatterplots of the NRS pain score vs fatigue.

**Supplementary Table S1.** Results from the simple model for direct association between hip OA pain and fatigue (continuous variable) adjusted for age, sex, and BMI.

| Model | Regression weight for a | 95% confidence interval | p-value | χ ^2^/df | RMSEA | CFI | Hoelter 0.05 index |
| --- | --- | --- | --- | --- | --- | --- | --- |
| Baseline | -1.44 | -2.58, -0.33 | 0.01 | 1.99/3 | <0.01 | 1.00 | 665 |
| T2 | -1.79 | -2.99, -0.59 | <0.01 | 3.20/3 | 0.02 | 0.99 | 413 |
| T5 | -1.97 | -3.03, -0.91 | <0.01 | 3.28/3 | 0.02 | 0.98 | 403 |
| T8 | -1.94 | -3.16, -0.75 | <0.01 | 2.93/3 | <0.01 | 1.00 | 452 |
| T10 | -1.84 | -3.00, -0.68 | <0.01 | 4.53/3 | 0.05 | 0.91 | 292 |

**Supplementary Table S2.** Results from testing the longitudinal mediating effect using hip pain at time-1, nocturnal pain at time, fatigue at time+1, adjusted for age, sex, and BMI.

| Model (n=170)  (time-1 > time > time+1) | Regression weight for a (p-value)  (95%CI) | Regression weight for b (p-value) (95%CI) | Regression weight for c (p-value)  (95%CI) | χ ^2^/df | RMSEA | CFI | Hoelter 0.05 index |
| --- | --- | --- | --- | --- | --- | --- | --- |
| T0 -> T2 -> T5 | -1.27 (p=0.06)  (-2.58, 0.04) | 0.04 (p=0.03)  (<0.01, 0.08) | 0.62 (p=0.85)  (-5.99, 7.23) | 2.00/3 | <0.01 | 1.00 | 660 |
| T2 -> T5 -> T8 | -0.73 (p=0.27)  (-2.00, 0.54) | 0.05 (p<0.01)  (0.01, 0.09) | -6.55 (p=0.05)  (-13.02, -0.08) | 3.15/3 | 0.02 | 1.00 | 420 |
| T5 ->T8 -> T10 | -0.66 (p=0.29)  (-1.89, 0.57) | 0.04 (p<0.01)  (0.02, 0.06) | -3.06 (p=0.44)  (-10.78, 4.66) | 3.16/3 | 0.02 | 0.99 | 418 |
| T0 -> T2 -> T8 | -1.53 (p=0.02)  (-2.80, -0.26) | 0.04 (p=0.03)  (<0.01, 0.08) | 1.78 (p=0.59)  (-4.63, 8.19) | 1.97/3 | <0.01 | 1.00 | 670 |
| T0-> T2 -> T10 | -2.03 (p<0.01)  (-3.38, -0.68) | 0.03 (p=0.03)  (-<0.01, 0.07) | -1.17 (p=0.73)  (-8.51, 4.97) | 1.99/3 | <0.01 | 1.00 | 665 |
| T0 -> T5 -> T8 | -1.18 (p=0.07)  (-2.45, 0.09) | 0.04 (p=0.01)  (<0.01, 0.08) | -6.50 (p=0.05)  (-13.00, 0.01) | 1.97/3 | <0.01 | 1.00 | 670 |
| T0-> T5 -> T10 | -1.94 (p<0.01)  (-3.39, -0.59) | 0.04 (p=0.01)  (<0.01, 0.08) | -3.60(p=0.31)  (-10.58, 3.38) | 1.99/3 | <0.01 | 1.00 | 666 |
| T0 -> T8-> T10 | -2.06 (p<0.01)  (-3.39, -0.73) | 0.02 (p=0.32)  (-0.37, 0.41) | -2.85 (p=0.44)  (-10.02, 4.32) | 2.14/3 | <0.01 | 1.00 | 619 |
| T2 ->T5-> T10 | -1.25 (p=0.08)  (-2.64, 0.14) | 0.05 (p<0.01)  (0.01, 0.09) | -4.65 (p=0.20)  (-11.80, 2.50) | 3.16/3 | 0.02 | 1.00 | 418 |
| T2 ->T8 -> T10 | -1.37 (p=0.05)  (-2.74, <0.01 | 0.02 (p=0.19)  (-0.02, 0.06) | -2.49 (p=0.51)  (-9.88, 4.90) | 3.20/3 | 0.02 | 0.99 | 414 |

**Supplementary Table S3.** Results from testing the mediating effect of nocturnal pain on the association

between hip OA pain and fatigue (continuous variable) adjusted for age, sex, and BMI for only those who did not received a hip replacement (n=132).

| Model  (n=132) | Regression weight for a (p-value)  (95%CI) | Regression weight for b (p-value)  (95%CI) | Regression weight for c (p-value)  (95%CI) | χ ^2^/df | RMSEA | CFI | Hoelter 0.05 index |
| --- | --- | --- | --- | --- | --- | --- | --- |
| Baseline | -1.28 (p=0.08)  (-2.71, 0.15) | 0.08 (p<0.01)  (0.04, 0.12) | -0.20 (p=0.95)  (-6.32, 5.92) | 0.99/3 | <0.01 | 1.00 | 1037 |
| T2 | -2.51 (p<0.01)  (-4.20, -0.82) | 0.09 (p<0.01)  (0.05, 0.13) | 1.17 (p=0.75)  (-6.02, 8.36) | 1.34/3 | <0.01 | 1.00 | 766 |
| T5 | -1.54 (p=0.04)  (-2.99, -0.09) | 0.08 (p<0.01)  (0.04, 0.12) | -4.89 (p=0.23)  (-12.81, 3.03) | 2.24/3 | 0.02 | 1.00 | 458 |
| T8 | -1.97 (p=0.01)  (-3.34, -0.60) | 0.07 (p<0.01)  (0.03, 0.11) | -2.74 (p=0.46)  (-9.95, 4.47) | 1.47/3 | <0.01 | 1.00 | 699 |
| T10 | -1.01 (p=0.17)  (-2.44, 0.42) | 0.07 (p<0.01)  (0.03, 0.11) | -5.57 (p=0.18)  (-13.68, 2.54) | 3.35/3 | 0.03 | 0.99 | 306 |

**Supplementary Table S4.** Results from testing the mediating effect of nocturnal pain on the association between hip OA pain and fatigue (continuous variable) adjusted for age, sex, and BMI for those with hip pain (and possible knee pain n=588). The prevalence of nocturnal pain (baseline) is 38%.

| Model  N=588 | Regression weight for a (p-value)  (95%CI) | Regression weight for b (p-value)  (95%CI) | Regression weight for c (p-value)  (95%CI) | χ ^2^/df | RMSEA | CFI | Hoelter 0.05 index |
| --- | --- | --- | --- | --- | --- | --- | --- |
| Baseline | -1.41 (p<0.01)  (-2.10, -0.72) | 0.10 (p<0.01)  (0.08, 0.12) | -1.88 (p=0.23)  (-4.94, 1.18) | 1.33/3 | <0.01 | 1.00 | 4046 |
| T2 | --1.51 (p<0.01)  (-2.24, -0.78) | 0.08 (p<0.01)  (0.06, 0.10) | -1.84 (p=0.31)  (-5.37, 1.69) | * |  |  |  |
| T5 | - 1.48 (p<0.01)  (-2.13, -0.83) | 0.09 (p<0.01)  (0.07, 0.11) | -5.00 (p=0.01)  (-8.67, -1.33) | 3.02/3 | <0.01 | 1.00 | 1521 |
| T8 | -1.82 (p=0.01)  (-2.51, -1.13) | 0.08 (p<0.01)  (0.06, 0.10) | -4.53 (p=0.02)  (-8.37, -0.79) | 1.75/3 | <0.01 | 1.00 | 2625 |
| T10 | -1.53 (p<0.01)  (-2.20, -0.86) | 0.09 (p<0.01)  (0.07, 0.11) | -4.60 (p=0.02)  (-8.44, -0.76) | 2.82/3 | <0.01 | 1.00 | 1629 |

*The saturated model was not fitted to the data of at least one group; chi square statistic and other fit measures are not reported.

**Supplementary Table S5.** Results from testing the longitudinal mediating effect of nocturnal pain on the association between hip OA pain and fatigue level for those with hip pain (and possible knee pain n=588).

| Model n=588  (time-1 > time > time+1) | Regression weight for a (p-value) (95%CI) | Regression weight for b (p-value) (95%CI) | Regression weight for c (p-value) (95%CI) | χ ^2^/df | RMSEA | CFI | Hoelter 0.05 index |
| --- | --- | --- | --- | --- | --- | --- | --- |
| T0 -> T2 -> T5 | -1.19 (p=0.01)  (-1.95, -0.43) | 0.05 (p<0.01)  (0.03, 0.07) | -3.87 (p=0.05)  (-7.40, -0.34) | -9,5^14^/3 | <0.01 | 1.00 | NA |
| T2 -> T5 -> T8 | -1.52 (p=<0.01)  (-2.25, -0.79) | 0.06 (p<0.01)  (0.04, 0.08) | -4.15 (p=0.02)  (-7.68, -0.62) | 4.33/3 | 0.03 | 0.99 | 1060 |
| T5 ->T8 -> T10 | -0.98 (p<0.01)  (-1.61, -0.35) | 0.05 (p=<0.01)  (0.03, 0.07) | -5.49 (p<0.01)  (-9.10, -1.88) | 3.15/3 | 0.01 | 1.00 | 1456 |
| T0 -> T2 -> T8 | -1.19 (p<0.01)  (-1.95, -0.43) | 0.05 (p<0.01)  (0.03, 0.07) | -3.87 (p=0.03)  (-7.40, -0.34) | 1.16/3 | <0.01 | 1.00 | 3960 |
| T0-> T2 -> T10 | -1.58 (p<0.01)  (-2.32, -0.84) | 0.05 (p<.0.01)  (0.03, 0.07) | -1.62 (p=0.36)  (-5.07, 1.83) | 1.18/3 | <0.01 | 1.00 | 3896 |
| T0 -> T5 -> T8 | -1.14 (p<0.01)  (-1.90, -0.38) | 0.04 (p<0.01)  (0.02, 0.06) | -5.23 (p<0.01)  (-8.70, -1.76) | 1.15/3 | <0.01 | 1.00 | 3989 |
| T0-> T5 -> T10 | -1.52 (p<0.01)  (-2.26, -0.78) | 0.04 (p<0.01)  (0.02, 0.06) | -3.51 (p=0.04)  (-6.92, -0.10) | 1.17/3 | <0.01 | 1.00 | 3936 |
| T0 -> T8-> T10 | -1.44 (p<0.01)  (-2.18, -0.70) | 0.04 (p<0.01)  (0.02, 0.06) | -5.90 (p<0.01)  (-9.37, -2.43) | 1.20/3 | <0.01 | 1.00 | 3830 |
| T2 ->T5-> T10 | -1.36 (p<0.01)  (-2.09, -0.63) | 0.06 (p<0.01)  (0.04, 0.08) | -3.07 (p=0.09)  (-6.58, 0.44) | 4.33/3 | 0.01 | 0.99 | 1061 |
| T2 ->T8 -> T10 | -1.24 (p<0.01)  (-1.95, -0.53) | 0.05 (p<0.01)  (0.03, 0.07) | -5.51 (p<0.01)  (-6.22, -4.81) | 4.30/3 | 0.03 | 0.99 | 1067 |

**Supplementary Table S6**: Results from testing the mediating effect of nocturnal pain on the association between hip OA pain and fatigue (continuous variable) adjusted for age, sex, Kellgren and Laurence score ≥2 any hip and BMI. The estimations are presented with p-value.

| Model  (n=170) | Regression weight for a (p-value) | Regression weight for b (p-value) | Regression weight for c (p-value) | χ ^2^/df | RMSEA | CFI | Hoelter 0.05 index |
| --- | --- | --- | --- | --- | --- | --- | --- |
| Baseline | -1.45 (p=0.02) | 0.07 (p<0.01) | -1.72 (p=0.54) | * |  |  |  |
| T2 | -1.73 (p=0.01) | 0.08 (p<0.01) | 1.14 (p=0.73) | -2.5x10^14^/6 | <0.01 | 1.00 | - |
| T5 | -1.45 (p=0.02) | 0.08 (p<0.01) | -6.35 (p=0.08) | -2.5x10^14^/6 | <0.01 | 1.00 | - |
| T8 | -1.95 (p<0.01) | 0.06 (p<0.01) | -3.15 (p=0.38) | -2.4x10^14^/6 | <0.01 | 1.00 | - |
| T10 | -1.53 (p=0.02) | 0.08 (p<0.01) | -4.43 (p=0.26) | -2.4x10^14^/6 | <0.01 | 1.00 | - |

*The saturated model was not fitted to the data of at least one group; chi square statistic and other fit measures are not reported.

**T0
n= 170**

**T2
n= 158**

**T5
n= 151**

**T8
n= 145**

**T10
n= 143**

**Missing**

Temporary: n= 5
Permanent: n= 7

**Missing**

Temporary: n= 4
Permanent: n= 8

**Missing**

Temporary: n= 4
Permanent: n=6

**Missing**

Temporary: n=1
Permanent: n=7

**Supplementary Figure S1.** Flow-chart indicating the available data per time point.


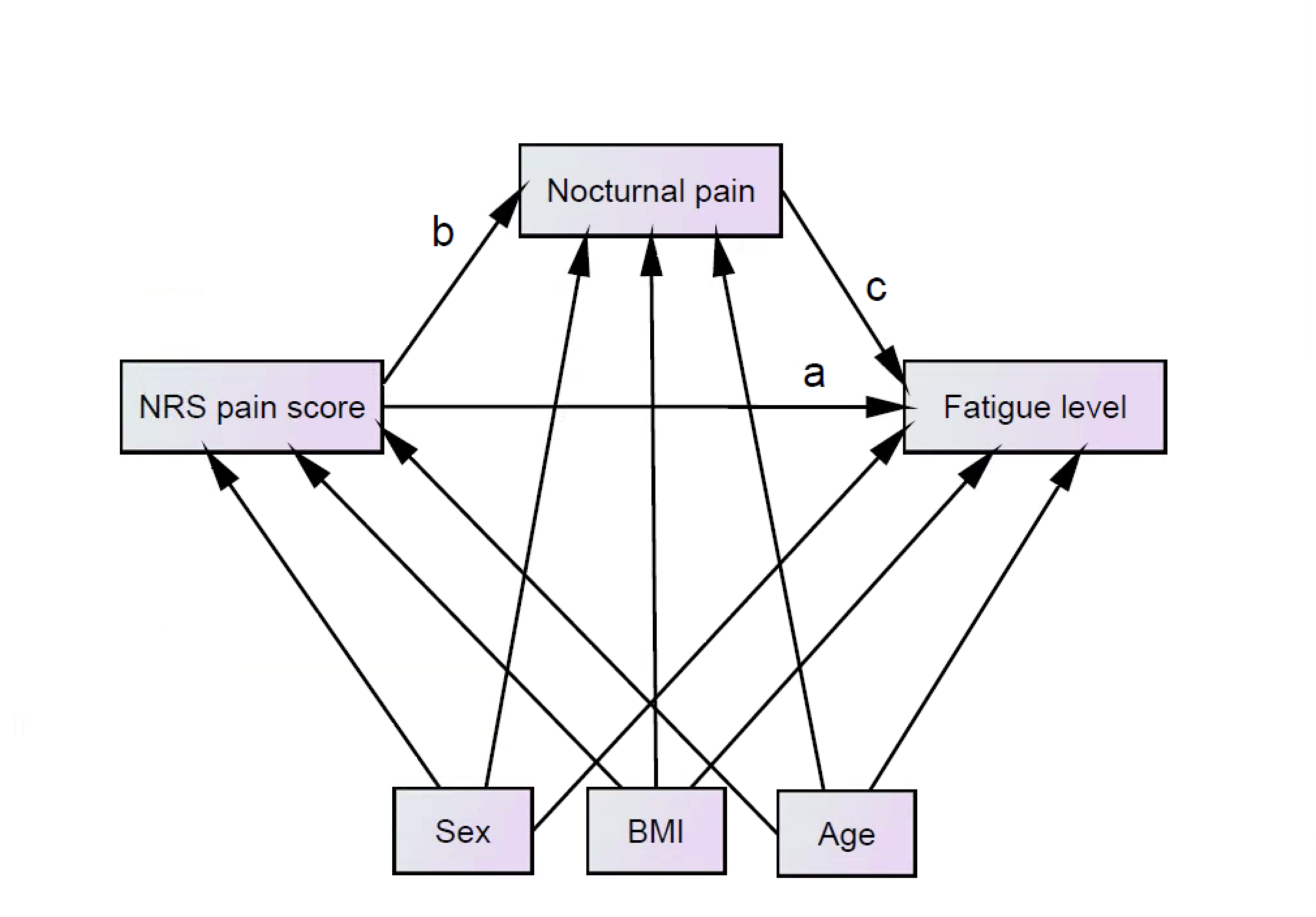


**Supplementary Figure S2. Testing for the longitudinal mediating effect using hip pain at time-1, nocturnal pain at time, fatigue at time+1, adjusted for confounders (sex, BMI, and age).** This model is applicable for NRS pain score at baseline (time-1), Nocturnal pain at T2 (time), fatigue at T5 (time+1) and all the confounders at baseline. The figure can be applied in the same way for the other models as shown in Supplementary Table S1. The estimates are presented in Supplementary Table S1 for the outcomes a, b and c.


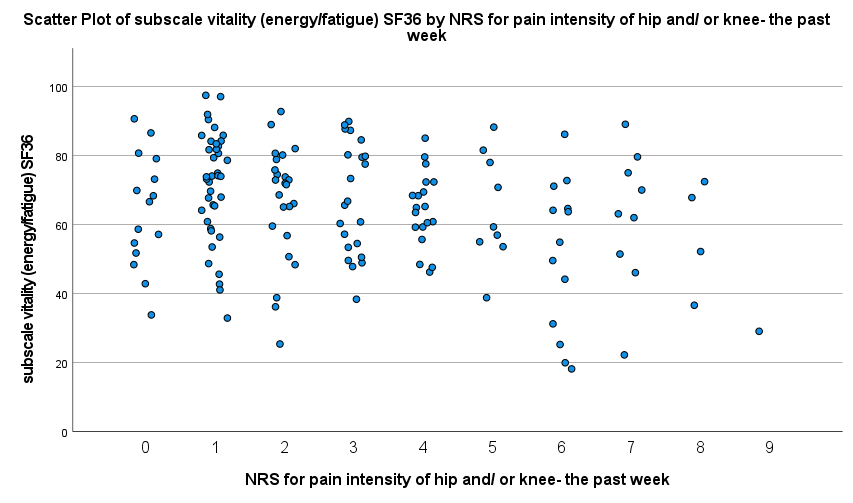

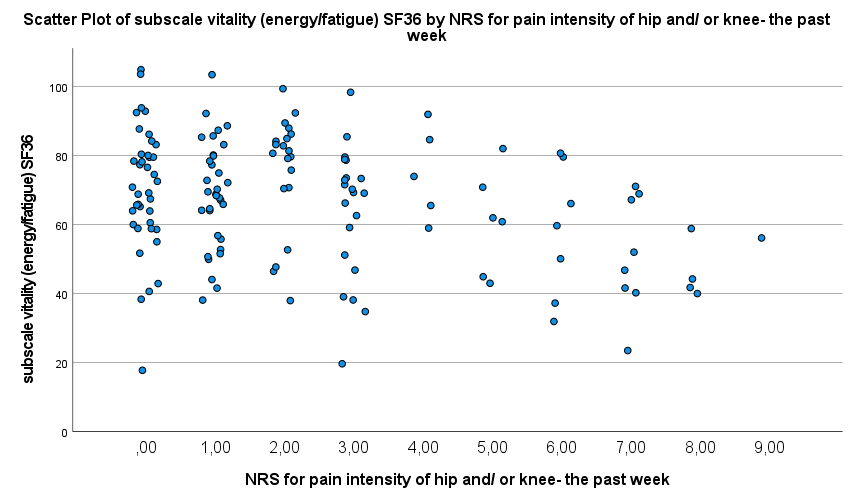

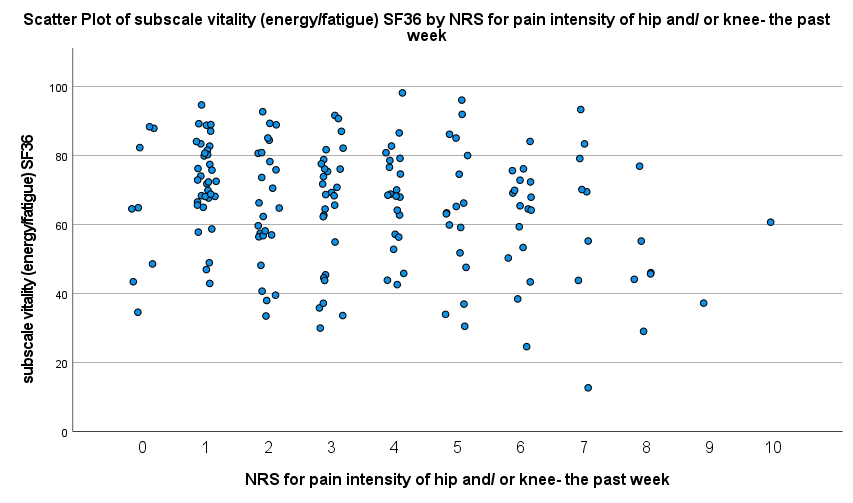

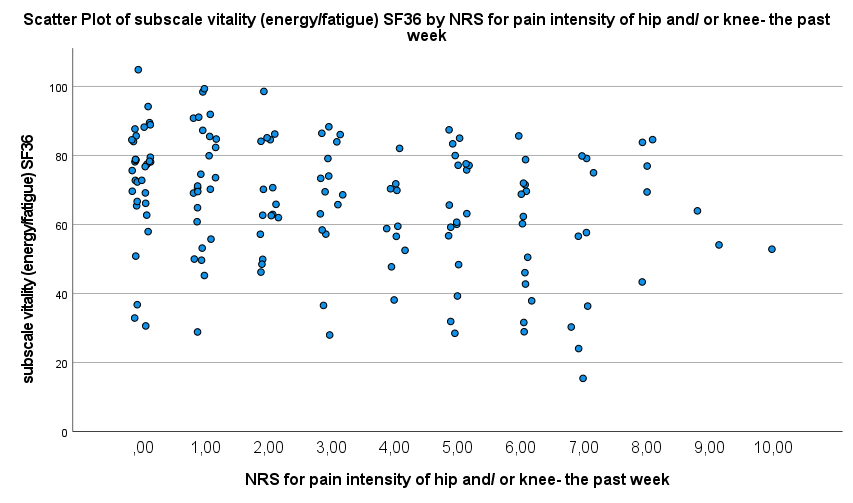

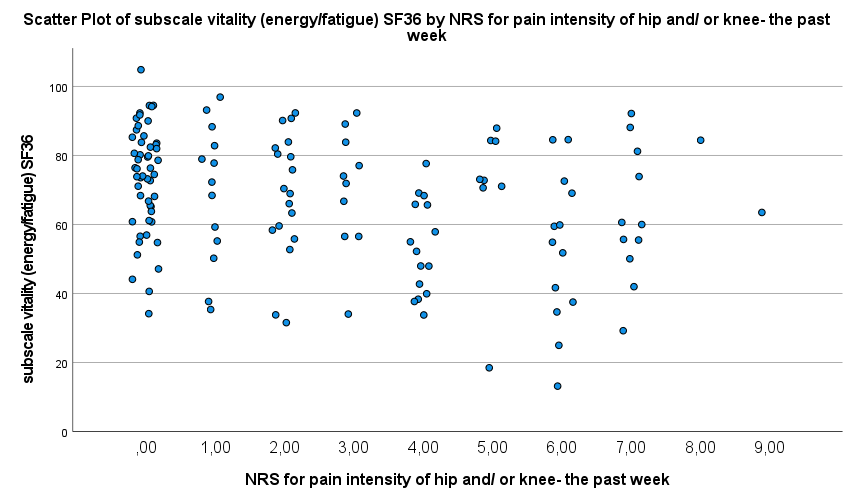


**Supplementary Figure S3.** Scatterplots of the NRS pain score vs fatigue (upper left = baseline; upper right = t2; middle left= t5; middle right =t8; lower left = t10).
